# A New Approach to Fall Detection Based on Improved Dual Parallel Channels Convolutional Neural Network

**DOI:** 10.3390/s19122814

**Published:** 2019-06-24

**Authors:** Xiaoguang Liu, Huanliang Li, Cunguang Lou, Tie Liang, Xiuling Liu, Hongrui Wang

**Affiliations:** 1 College of Electronic and Information Engineering, Hebei University, Baoding 071002, China; lxg_hbu@163.com (X.L.); libigfat@163.com (H.L.); loucunguang@163.com (C.L.); lanswer@163.com (T.L.); htw_hbu@163.com (H.W.); 2 Key Laboratory of Digital Medical Engineering of Hebei Province, Hebei University, Baoding 071002, China

**Keywords:** fall detection, surface electromyography, spectrograms, convolutional neural network, pattern recognition

## Abstract

Falls are the major cause of fatal and non-fatal injury among people aged more than 65 years. Due to the grave consequences of the occurrence of falls, it is necessary to conduct thorough research on falls. This paper presents a method for the study of fall detection using surface electromyography (sEMG) based on an improved dual parallel channels convolutional neural network (IDPC-CNN). The proposed IDPC-CNN model is designed to identify falls from daily activities using the spectral features of sEMG. Firstly, the classification accuracy of time domain features and spectrograms are compared using linear discriminant analysis (LDA), k-nearest neighbor (KNN) and support vector machine (SVM). Results show that spectrograms provide a richer way to extract pattern information and better classification performance. Therefore, the spectrogram features of sEMG are selected as the input of IDPC-CNN to distinguish between daily activities and falls. Finally, The IDPC-CNN is compared with SVM and three different structure CNNs under the same conditions. Experimental results show that the proposed IDPC-CNN achieves 92.55% accuracy, 95.71% sensitivity and 91.7% specificity. Overall, The IDPC-CNN is more effective than the comparison in accuracy, efficiency, training and generalization.

## 1. Introduction

A fall is an accidental injury that is very common in everyday life. According to the latest statistics of the China Office for Ageing, as of the end of 2017, the number of elderly people over the age of 60 reached 240 million, accounting for 17.3% of the total population. This shows that China’s aging is more serious. In daily life, due to factors such as weak physical fitness and decreased balance, the elderly are extremely prone to fall. If not treated in time, it will cause serious harm to the elderly and even be life-threatening [1]. According to the "China Injury Prevention Report,” the incidence of falls in the elderly is 20.7% in China. Therefore, falls are the main cause of death, disability and loss of independence for people over 65 years old—about one in every three people. Therefore, fall detection is of great significance for improving the level of care for the elderly [2].

At present, the related research on human fall detection is mainly divided into two categories. One type is fall recognition based on video images. Sase, P.S. et al. [3] set the threshold to detect the falling action by extracting valid frames in video, filtering, binarization and connection components. Li, X. et al. [4] proposed a fall detection platform based on Kinect and support vector machine (SVM). Based on the logic rule and D-S evidence fusion method, the data were merged to identify the fall action and the accuracy rate was significantly improved. Baldewijns, G. et al. [5] proposed a delay fusion technique to improve the accuracy of fall detection. Combining the confidence of different video fall detection systems, four different aggregation methods were compared based on the lower area of the precision recall curve. Although this type of fall recognition has a better accuracy, it has a high requirement for the surrounding environment and a limited range of activities.

The other type is fall recognition based on various sensors including triaxial acceleration, acoustic sensors and new wearable sensors. Wang, F.T. et al. [6] proposed two new inertial parameters—acceleration cube product and angular velocity cube product—to distinguish between falls and other daily activities to improve the specificity of fall detection. Mao, A. et al [7] proposed a fall detection algorithm based on acceleration signal and Euler angle. To achieve better fall detection accuracy and convenience, the optimal position of the sensor placement is verified by comparing the detection performance of different positions of the human body. Er, P.V. et al. [8] used sound sensors to detect the sound pressure generated by falls and developed a fall detection algorithm based on fuzzy logic in conjunction with the accelerometer, which was used to process sound pressure and acceleration signals, effectively improving the accuracy of fall recognition. Hsieh, C.Y. et al. [9] proposed a new layered fall detection method based on the multiphase drop model and combined it with the feature extraction mode based on threshold value to effectively identify fall events from continuous sensor data. Mezghani, N. et al. [10] proposed a new fall detection system based on intelligent textiles, which uses nonlinear support vector machine to determine the fall direction, which is helpful for studying the impact of falling according to the fall direction. This kind of fall recognition has been applied in a wider range and its real-time detection and portability have been further improved. However, due to the relatively small amount of information contained in the sensor signals, the accuracy of fall recognition has been relatively reduced.

Common methods for fall detection include SVM [11,12], artificial neural network (ANN) [13,14], hidden Markov model [15] and decision tree [16] and so forth. Convolutional neural network (CNN) is a deep feedforward neural network that has been developed in recent years and has attracted extensive attention. It is most commonly used in supervised learning problems in the image field, such as image recognition and computer vision. As early as 1989, LeCun et al. [17] proposed the initial convolutional neural network model and improved it later. After Alex Net [18] won the 2012 ImageNet competition, convolutional neural networks have become synonymous with deep learning in the field of image recognition and there are more and more applications in other fields. At present, Hu, Y. et al. [19] proposed a Convolution-Long Short-Term Memory (Conv-LSTM) structure based on the attention mechanism to better capture the time characteristics of surface electromyography (sEMG) signals to solve the problem of gesture recognition.

The work presented in this paper addresses these severe limitations while still achieving state of the art results. The approach is based on employing improved dual parallel channels convolutional neural network (IDPC-CNN) to perform the classification of spectrograms of the sEMG in order to achieve fall detection. The original sEMG data is collected from the rectus femoris, medial femoral muscle, tibialis anterior muscle and gastrocnemius muscle. Firstly, sEMG is pre-processed to extract effective information. Sliding hamming window [20] is used to obtain the spectrum image features of sEMG and the spectrum image was further dimensionally reduced. The dimensionality reduction spectra [21] is used as the input of IDPC-CNN to perform the fall motion recognition. Compared with other identification methods, the proposed method has the advantages of low equipment price, high recognition accuracy, large operating range and protection of privacy. Overall, the method has higher accuracy, more efficient training and greater generalization. Falling down on the old man’s injury is closely related to whether he can get timely treatment after the fall. This method can detect the occurrence of the fall action more quickly and then issue an alarm to help the elderly get help faster.

## 2. SEMG Signal Acquisition and Preprocessing

### 2.1. SEMG Acquisition

**Figure 1:**
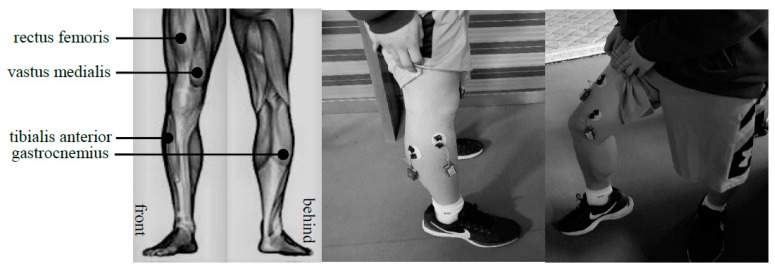
The position of surface electromyography (sEMG) electrode.

There are many muscles that control the flexion and extension of the legs. In this paper, four main muscles of the rectus femoris, medial femoral muscle, tibialis anterior muscle and gastrocnemius muscle are selected as the collection objects. The corresponding electrode positions of each muscle are shown in Figure 1.

**Figure 2:**
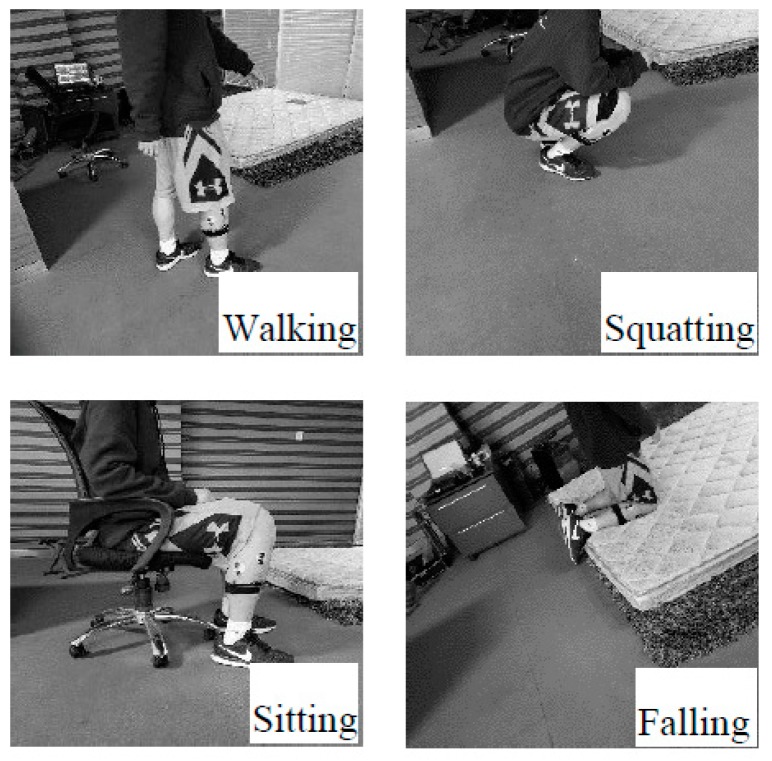
The 4 gestures considered in this work.

The sEMG data are collected by the data transmission system (DTS) series wireless telemetry surface electromyography acquisition system produced by NORAXON, USA. It supports up to 16 channels of sEMG simultaneously. The DTS provides a sEMG sampling frequency of 1500 Hz per channel. Five healthy men and five healthy women are selected as volunteers. The average age of the volunteers is 24. The volunteers did not exercise vigorously one week before the experiment to avoid the effect of muscle fatigue on the experiment. Before placing the electrodes on the skin, the body hair was removed from the measuring area and it was wiped with an alcohol cotton ball to remove dead skin. The preparatory work of the skin can minimize the interference with the measurement of the data. With the consent of the subjects and the subjects signed a written informed consent and privacy agreements before participating in the experiment. Volunteers agreed to use personal data in the context of medical, teaching and medical research and the dataset is freely distributed. Basic information and medical history of the 10 subjects are shown in Table 1:

Since the final purpose of this experiment is to distinguish between falls and daily actions, four different gestures are required. The classes are: walking, squatting, sitting and falling. The walking movement is completed on a flat road with a length of 10 m and the walking time is about 12 s. Data under the state of smooth walking are selected. Figure 2 shows the different gestures.

As stated previously, our experiment uses 4 sEMG channels, each sampled at 1500 Hz. During the data collection process, each movement was repeated 10 times. The subjects were required to rest for 10 s after completing one movement to avoid inaccurate data caused by muscle fatigue. A total of 400 sets of effective experimental data were obtained, including walking, squatting, sitting and falling, among which 100 sets were falling and 300 sets were the other activities. Each set of data contains 4 channel sEMG signals.

### 2.2. Signal Preprocessing

#### 2.2.1. Signal Denoising

**Figure 3:**
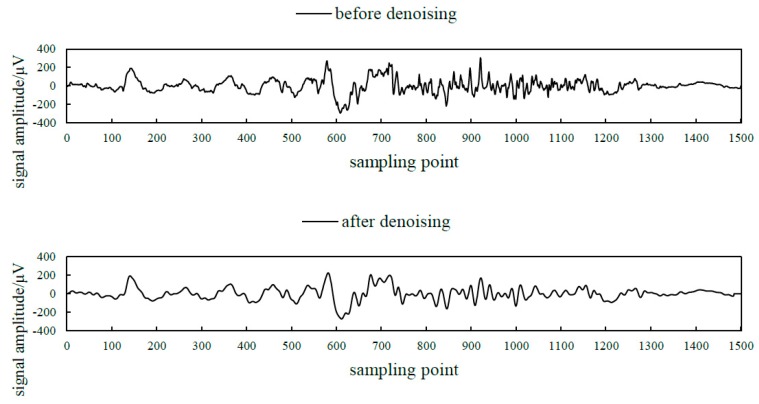
The comparison of sEMG signals before and after denoising.

The sEMG signal is a weak signal with a low frequency range of 10–500 Hz [22], so the original sEMG signal needs to be pre-processed. Since the DTS system provides a voltage interference shielding module, the 50 Hz power frequency notch processing was eliminated, thus ensuring the integrity of the original signal [23]. The third-order Butterworth low-pass filter and the third-order Butterworth high-pass filter were cascaded into a band-pass filter [24]. The cut off frequency of the low-pass filter was 500 Hz. The cut off frequency of the high pass filter was 10 Hz. The attenuation rate of the filter was 18 db per octave. Noise outside the effective frequency range was removed. The comparison of sEMG signals before and after denoising is shown in Figure 3.

#### 2.2.2. Extraction of Effective Signal Segment

There are some differences in the way muscles contract when different volunteers perform the same action [25]. Even the same volunteer cannot completely repeat the same action. This leads to a certain difference in the length and amplitude of the sEMG signal under the same action. In order to improve the motion recognition rate and reduce the amount of processed data, our system uses the sliding window energy threshold method to automatically extract each valid segment of the surface EMG signal [26].

Let $x_{ki}$ is the ith sampling point of the k-segment signal, the mean short-term energy of n sampling points after $x_{ki}$ is expressed as:

(1) $$E_{k}=\frac{1}{n}\sum_{i=1}^{n}x_{ki}^{2}$$

**Figure 4:**
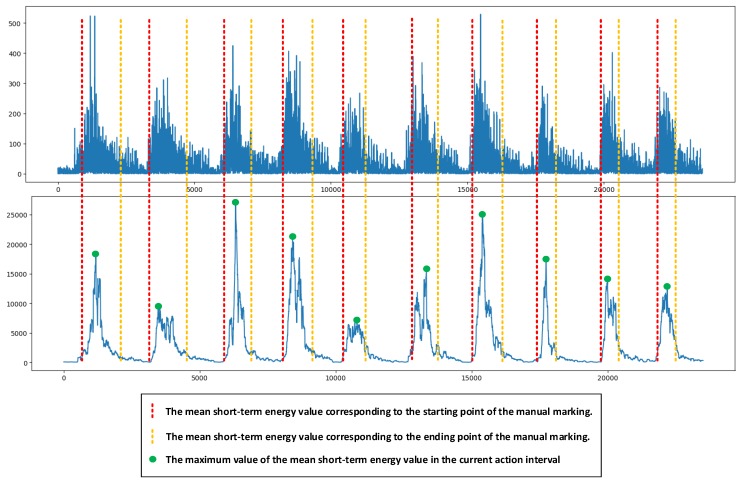
Mean short-term energy value result.

In order to divide the effective signal segment more accurately, set n to 100. The result of the signal passing through the sliding window to calculate the mean short-term energy is shown in Figure 4.

Partial data of the subjects were selected for manual segmentation of the action interval and the mean value of the ratio between the mean short-term energy corresponding to all starting points ($E_{k-start}$) and the maximum value of the mean short-term energy in the corresponding interval was calculated and multiplied by the maximum value of the mean short-term energy ($E_{k-max}$) of the rest data of the subjects, so as to obtain the static threshold of the starting point for the subject. The static threshold of the starting point (STS) was defined as:

(2) $$STS=Mean\left(\sum \frac{E_{k-start}}{Max\left(E_{k}\right)}\right)\cdot \;E_{k-max}$$

The mean value of the ratio between the mean short-term energy corresponding to all ending points ($E_{k-end}$) and the maximum value of the mean short-term energy in the corresponding interval was calculated and multiplied by the maximum value of the mean short-term energy ($E_{k-max}$) of the rest data of the subjects, so as to obtain the static threshold of the ending point for the subject. The static threshold of the ending point (STE) was defined as:

(3) $$STE=Mean\left(\sum \frac{E_{k-end}}{Max\left(E_{k}\right)}\right)\cdot \;E_{k-max}$$

**Figure 5:**
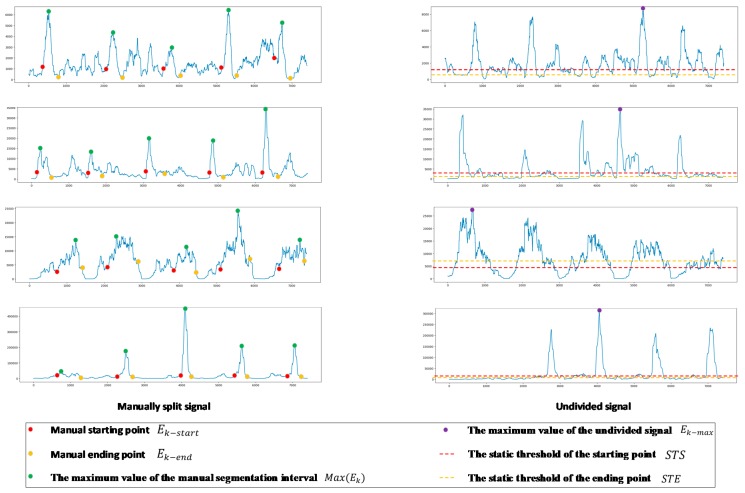
Threshold segmentation diagram.

The threshold calculation of the starting point and the ending point is shown in Figure 5.

When $E_{k}>STS$ and the mean value of the first l mean short-term energy should be less than the mean short-term energy of the current window, the value of l is related to the window size n and sampling frequency $f_{s}$:

(4) $$l=\frac{f_{s}}{2n}$$

Meanwhile, the mean value of the latter m mean short-term energy should be greater than the static threshold, the value of m is related to the window size n and sampling frequency $f_{s}$:

(5) $$\frac{f_{s}}{2n}<m<\frac{f_{s}}{n}$$

When $E_{k}<STE$ and the mean value of the latter l mean short-term energy should be less than the mean short-term energy of the current window.

**Figure 6:**
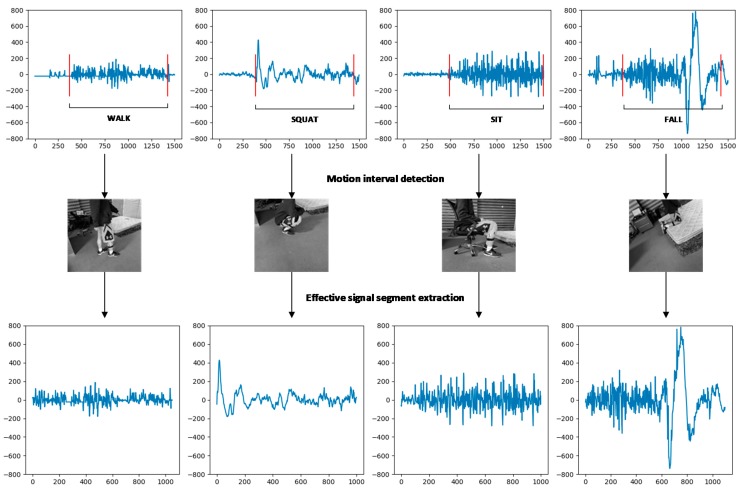
Effective signal segment extraction.

As mentioned earlier, in order to ensure that the extracted effective active segment had practical significance, the static thresholds STS and STE were set due to the difference between the movement and the muscle [27]. The method for extracting the active segment of the signal helps to improve the accuracy of the subsequent classification by reducing the interference information due to factors such as motion differences, muscle jitter and environmental influences [28]. The extraction of valid signal segments is shown in Figure 6.

## 3. SEMG Feature Selection and Fall Detection Method

### 3.1. SEMG Spectrograms Extraction

In order to avoid the frequency leakage caused by using a rectangular window to extract the sEMG spectrogram directly, the sliding Hamming window was selected to perform fast Fourier transform (FFT) on the sEMG. The window function of the Hamming window is:

(6) $$W\left(n,a\right)=\left(1-a\right)-a\cos\begin{array}{c} \left(2\pi \frac{n}{N-1}\right),\\ 0\leq n\leq N-1 \end{array}$$

In general, a is 0.46. Since the amplitude-frequency characteristic of the Hamming window is that the side lobe attenuation is large, the main lobe peak and the first side-lobe peak attenuation can reach 40 dB. After windowing, the middle data information is obvious and the data information on both sides is attenuated. When moving the window, moving the 1⁄3 or 1⁄2 window, the attenuated data in the previous window re-emerged, so that the window sliding became relatively stable and the spectrum diffusion was reduced to the minimum.

In order to further reduce the delay of fall recognition and improve the efficiency of the algorithm, FFT was adopted in this paper to extract the features of the spectrum.

Firstly, a sliding hamming window was used to carry out FFT on the sEMG signals of each channel for extracting the spectrograms. The horizontal axis of the spectrum is the time direction and the vertical axis is the frequency direction. Since the effective information of the sEMG signal is mainly concentrated in the range of 10–500 Hz, the spectrograms were processed to retain the effective frequency segment.

**Figure 7:**
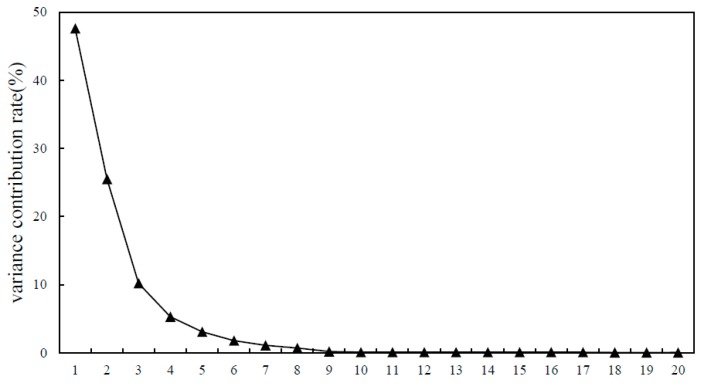
Variance contribution rate.

Since the different magnitude of sEMG channels, the spectrograms of sEMG were normalized to between 0 and 1 in order to improve the efficiency of the algorithm. Principal component analysis (PCA) was used to reduce the dimension of frequency direction of sEMG spectrogram data [29]. As can be seen from Figure 7 and Table 2, the cumulative variance contribution rate of the first eight principal components reached 95.3%. The reduced dimensional sEMG spectrum can be used to explain most of the effective information in the original high-dimensional sEMG spectrum, so as to adapt the data to the CNN classifier and reduce the classifier processing time.

**Figure 8:**
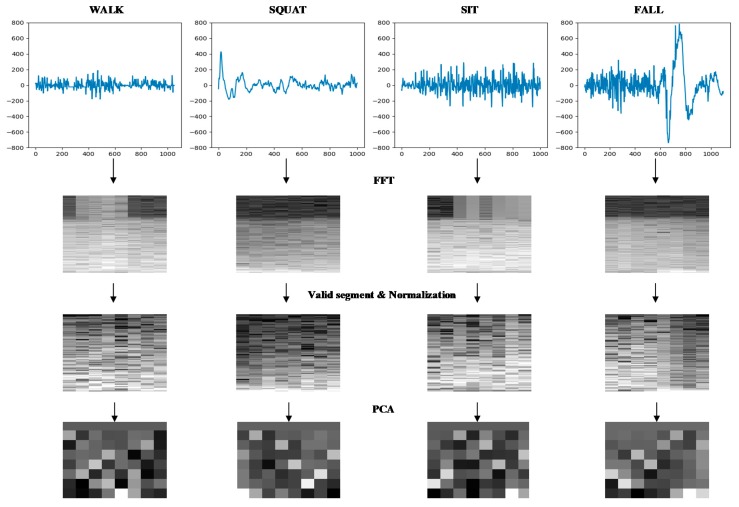
Spectrogram processing example.

The spectrum extraction and data dimensionality reduction for the four actions are shown in Figure 8.

**Figure 9:**
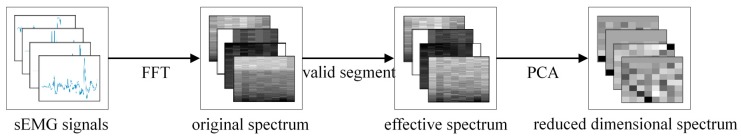
Feature extraction flow chart.

Using the above method, Figure 9 shows the spectrums extraction process of sEMG.

### 3.2. SEMG Feature Selection

**Figure 10:**
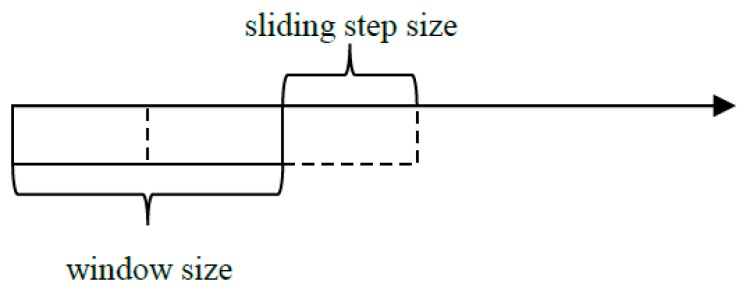
Sliding window schematic.

The beginning, process and end phases of an action contain different information. In order to effectively utilize the information, the sliding window is selected to extract the feature of sEMG signal. The sliding window consists of two key variables: the window size and the sliding step size [30], as shown in Figure 10.

In practical applications, the performance of the classifier should take priority over speed. In order to achieve an acceptable continuous classification, the latency should be less than 300 ms [31]. In our system, we opted for the windows of 200 ms (300 points) and the sliding step 100 ms (150 points). 

The features of sEMG are time-domain features, frequency domain features and time-frequency domain features. The linear discriminant analysis was used to compare the classification accuracy of time domain features and frequency domain features and the feature selection was further optimized. In the time domain, mean absolute value (MAV), variance (VAR), waveform length (WL), root mean square (RMS), zero crossing (ZC) and slope sign change (SSC) are selected. The calculation formula is as follows:

1. mean absolute value:
  (7) $$\mathrm{MAV}=\frac{1}{n}\sum_{i=1}^{n}\left|x_{ki}\right|$$
2. variance:
  (8) $$\mathrm{VAR}=\frac{1}{n}\sum_{i=1}^{n}{\left(x_{ki}-\bar{x_{k}}\right)}^{2}$$
3. waveform length:
  (9) $$\mathrm{WL}=\sum_{i=1}^{n-1}\left|x_{k\left(i+1\right)}-x_{ki}\right|$$
4. root mean square:
  (10) $$\mathrm{RMS}=\sqrt{\frac{1}{n}\sum_{i=1}^{n}x_{ki}^{2}}$$
5. zero crossing:
  (11) $$\mathrm{ZC}=\sum_{i=1}^{n-1}\begin{array}{c} {}[f\left(x_{ki}\times x_{k\left(i+1\right)}\right)\\ \cap \left|x_{ki}-x_{k\left(i+1\right)}\right|\geq \varepsilon ] \end{array}$$
6. slope sign change:
  (12) $$\mathrm{SSC}=\frac{1}{n-2}\sum_{i=2}^{n-1}f\left(i\right)$$
  (13) $$f\left(i\right)=\{\begin{array}{c} 0,\left(x_{ki}-x_{k\left(i-1\right)}\right)\left(x_{k\left(i+1\right)}-x_{ki}\right)>0\\ 1,\left(x_{ki}-x_{k\left(i-1\right)}\right)\left(x_{k\left(i+1\right)}-x_{ki}\right)<0 \end{array}$$

The frequency domain feature selects the spectrograms. The spectrograms contain two kinds of information, time and frequency, which reflects more information that cannot be observed in time domain features. The analysis of spectrogram analysis can improve the discriminability between different actions.

**Figure 11:**
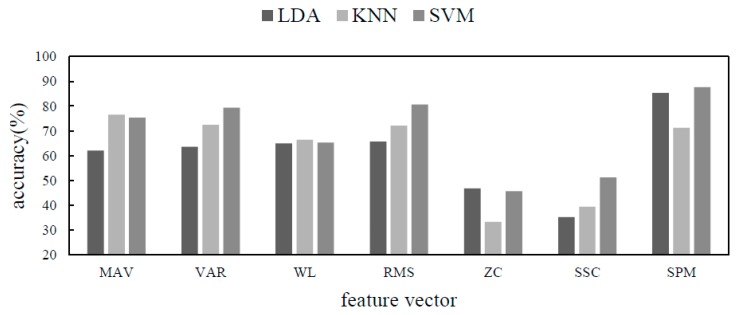
Accuracy comparison diagram.

Three kinds of classifiers, LDA, KNN and SVM, were selected to compare the performance of different features under different classifiers. The classification accuracy is shown in Figure 11.

It can be seen from the figure that when the spectrogram was used as the feature, the classification accuracy was greatly improved compared with several time domain features. Spectrograms do not perform well under KNN, a classifier that relies on distance-dependent classification but also reach normal levels. The comprehensive performance of SVM in the three classifiers was better. Among several time domain features, the comprehensive index of RMS was better. RMS and SPM were selected as features to further compare the accuracy under IDPC-CNN.

### 3.3. Fall Detection Method Based on IDPC-CNN

**Figure 12:**
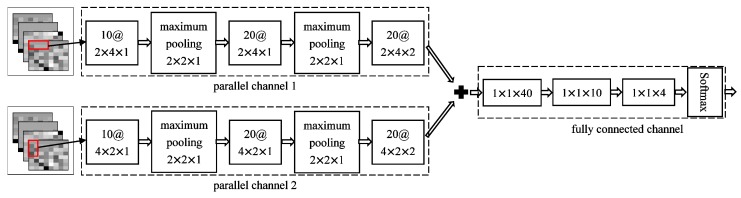
Improved dual parallel channels convolutional neural network structure.

The network structure of IDPC-CNN is shown in Figure 12. The first two convolutional layers of the parallel channel 1 adopted the third-order convolution kernels of 2 × 4 × 1. The difference is that the number of convolution kernels was 10 in first layer while the number was 20 in the second layer. Then the maximum pooling layer was added after each convolutional layer to improve the robustness of the algorithm and reduce the degradation of classification accuracy caused by local noise. In the last convolutional layer, the third-order convolution kernels of 2 × 4 × 2 was used and the number was 20. Similar to the first channel. The first two convolutional layers of parallel channel 2 adopted the third-order convolution kernels of 4 × 2 × 1 and the last convolutional layer adopted the third-order convolution kernels of 4 × 2 × 2. The number of convolution kernels of each layer was the same as the configuration of parallel channel 1. The rectangular convolution kernel was proposed, which can enhance parallel channel 1 for focusing on the time domain and the parallel channel 2 for focusing on the frequency domain and the square convolution kernel cannot reflect the focus on, such as single-channel CNN. Compared with the square convolution kernel, the rectangular convolution kernel can improve the information extraction of different features and network performance more effectively. The spectrums of different channels are mixed to detect the correlation between different muscles.

The focus of the two channels is different. Channel 1 used a 2 × 4 convolution kernel, which focused on analyzing horizontal continuous information in the spectrum, while channel 2 used a 4 × 2 convolution kernel, which focused on analyzing vertical continuous information in the spectrum. The two channels do not influence each other in the process of feature extraction. Finally, the outputs of the two channels were combined and passed to the fully connected channel.

The fully connected channel was composed of three fully connected layers and one softmax layer. The first fully connected layer has 40 units, the middle layer has 10 units and the last fully connected layer has 4 units, corresponding to the number of classification actions. In order to avoid gradient disappearance and overfitting problem the rectified linear unit (ReLU) was used as the activation function in the fully connected layer and the dropout with a probability of 0.5 was added between the fully connected layer to improve the training speed. The Softmax layer was used to convert the output to the probability distribution of different actions. The Softmax function formula was as follows:

(14) $$P\left(y=i|x\right)=\frac{e^{h\left(x,y_{i}\right)}}{\sum_{j=1}^{n}e^{h\left(x,y_{j}\right)}}$$

where $h\left(x,y_{i}\right)$ represents an original measure that input x belongs to class i and P(y=i|x) represents the probability that input x belongs to class i.

RMS and SPM were selected as features and IDPC-CNN as the classifier. The comparison test was conducted under the same experimental environment and the same data set. The CPU was Intel i5-8400 with 8GB DDR4 RAM and the GPU was NVIDIA GT710. All performance indicators were obtained through 10-fold cross-validation. The program running time of each stage and the final accuracy are shown in Table 3.

It can be seen from the table that the feature extraction time and the classifier training time of the SPM were greater than the RMS and the classifier test result time was not much different. Since the method is to detect the occurrence of the fall action more quickly and issue an alarm, the SPM was selected as the input feature in terms of classifier test result time and accuracy.

## 4. Experiment

In order to verify the effectiveness of the proposed method, support vector machine (SVM) and CNN [32] with several different structures were selected for comparative experiments.

1. SVM

The radial basis kernel function has the characteristics of simple structure and strong generalization ability and was selected as the kernel function.

2. DPC1-CNN

**Figure 13:**
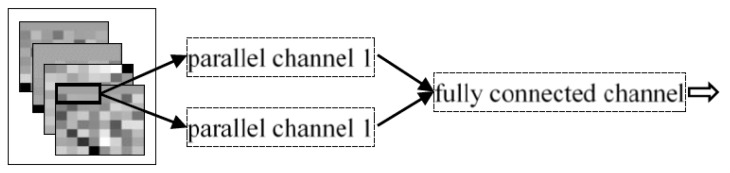
Dual parallel channel 1 convolutional neural network structure.

The structure of two parallel channels 1 was adopted and the fully connected channel consistent with IDPC-CNN, as shown in Figure 13.

3. DPC2-CNN

**Figure 14:**
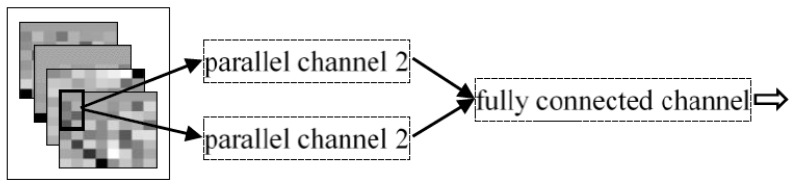
Dual parallel channel 2 convolutional neural network structure.

The structure of two parallel channels 2 was adopted, the fully connected channel consistent with IDPC-CNN, as shown in Figure 14.

4. Single channel CNN

**Figure 15:**
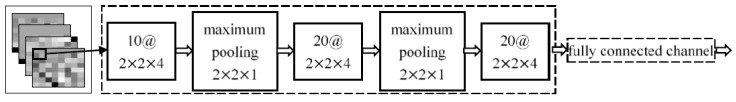
Single-channel convolutional neural network (CNN) structure.

The single-channel structure was adopted and the convolutional layer adopted the third-order convolution kernels of 2 × 2 × 4. Other parameters were the same as the IDPC-CNN proposed in this paper. The structure is shown in Figure 15.

The data set from all 10 people was trained by 10-fold cross-validation. In order to prevent each person’s data from being trained due to mixed data operation, the data set was divided into ten parts according to 10 people. The data of 9 people were used as the training set and the data of 1 person is used as the test set and the average value of the results of 10 times is taken as the estimation of algorithm performance. All the reported statistics are obtained with 10-fold cross-validation. Since the data of all training sets are sent to the CNN for iterative training at one time, the computational load is too large and the gradient descent is slow. In order to improve the training efficiency, the method of batch training is adopted. In each iteration, a batch of data in the training set is randomly selected and sent to the CNN, which can effectively improve the training speed, reduce the computation amount of each iteration, find the optimal gradient descent direction faster and avoid the problem of overfitting and gradient disappearance.

**Figure 16:**
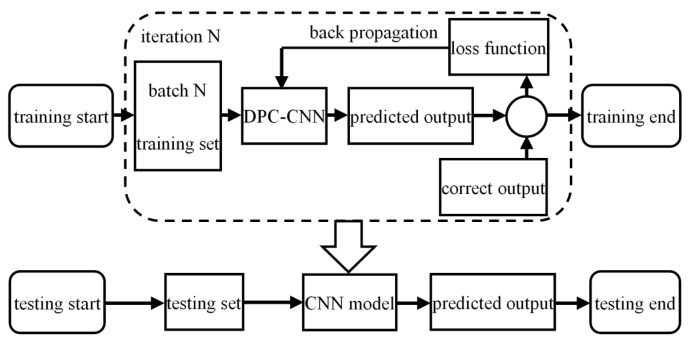
Train and test flow chart.

In order to reduce the amount of calculation and improve the training efficiency, the experiment uses a batch training method. A batch of data in the training set is randomly selected as the training data of the network in each iteration cycle. It can effectively improve the training speed, reduce the calculation amount of each iteration, find the optimal gradient descent direction more quickly to avoid the problem of overfitting and gradient disappearing. The train and test flow chart is shown in Figure 16:

In the same experimental environment and the same data set, the comparison test was carried out to analyze the accuracy of different methods for fall recognition. In order to quantitatively analyze the experimental results, fall recognition results were divided into four categories: TP-fall samples are identified as falls, FP-daily activity samples are identified as falls, TN-daily activity samples are identified as daily activities, FN-fall samples are identified as daily activities.

The following three performance indicators are used to judge the performance of the CNN and the SVM classifier:

1. Accuracy ($A_{c}$), the accuracy of all samples and the formula is:
  (15) $$A_{c}=\frac{TP+TN}{TP+FP+TN+FN}\times 100\%$$
2. Sensitivity ($S_{e}$), the detection rate of all fall samples and the formula is:
  (16) $$S_{e}=\frac{TP}{TP+FN}\times 100\%$$
3. Specificity ($S_{p}$), the detection rate of all daily activity samples and the formula is:
  (17) $$S_{p}=\frac{TN}{FP+TN}\times 100\%$$

**Figure 17:**
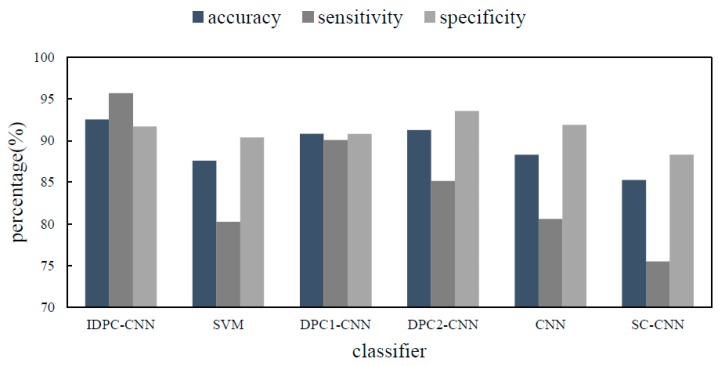
Performance index comparison.

The experimental results are shown in Figure 17.

According to the analysis of experimental results, it can be verified that the proposed sEMG fall warning method based on IDPC-CNN has better classification performance. 

## 5. Results

Aiming at the fall of the elderly, this paper proposes a fall detection method using sEMG based on IDPC-CNN. The method realizes the identification of three daily actions and forward falls using IDPC-CNN. This method has achieved an accuracy of 92.55%, a sensitivity of 95.71% and a specificity of 91.7%. The experimental results show that the method can realize the classification of four kinds of actions. In the next step, the parameters and structure of the convolutional neural network for fall recognition will be further optimized and the self-correction training will be considered to reduce the running time and improve the recognition accuracy.

## 6. Discussion

The IDPC-CNN classifier is significantly superior to other methods in terms of accuracy, sensitivity and specificity. Due to the small convolution kernel and cannot acquire more input information features, single channel CNN has the lowest performance indicators. The performance indexes of DPC1-CNN and DPC2-CNN classifiers are slightly lower than IDPC-CNN. It shows that the dual channel can improve the accuracy of classification by using different convolution kernels and combining the time direction and frequency direction of the spectrums. In addition to single channel CNN, the indicators of other methods are significantly better than that of SVM. Using the spectral information of sEMG, the improved dual parallel channel convolutional neural network updates the parameters by iterative training and back propagation, which effectively reduces the training time and improves the accuracy of the algorithm. The results show that the running time of online classification prediction is within the acceptable delay range. In the clinical field, it will have certain practical significance for the real-time warning of the fall for the elderly.

## Figures and Tables

**Table 1:** Basic information of different research subjects.

Subject	Gender	Age	Height/cm	Weight/kg	Lower Limbs Diseases
1	Male	24	176	74	No
2	Male	23	175	78	No
3	Male	23	172	72	No
4	Male	25	179	83	No
5	Male	24	170	70	No
6	Female	27	168	51	No
7	Female	23	165	47	No
8	Female	24	162	45	No
9	Female	24	170	55	No
10	Female	23	160	44	No

**Table 2:** Variance and cumulative variance contribution rate.

Principal Component	Variance Contribution Rate	Accumulated Variance Contribution Rate
1	47.6	47.6
2	25.5	73.1
3	10.2	83.3
4	5.3	88.6
5	3.1	91.7
6	1.8	93.5
7	1.1	94.6
8	0.7	95.3
9	0.2	95.5
10	0.1	95.6
20	0.06	96.38

**Table 3:** Performance indicators comparison.

Performance Indicators	RMS	SPM
Signal preprocessing time(ms)	25.09	25.09
Feature extraction time(ms)	101.37	357.16
Classifier training time(h)	7.5	10.6
Classifier test result time(ms)	57.12	63.5
Accuracy(%)	84.21	92.55
